# Line-field confocal optical coherence tomography for diagnosis and treatment monitoring of subtle basal cell carcinomas in a patient with nevoid basal cell carcinoma syndrome

**DOI:** 10.1016/j.jdcr.2026.07.011

**Published:** 2026-07-14

**Authors:** Mehdi Boostani, Cerrene N. Giordano, Gyorgy Paragh

**Affiliations:** Department of Dermatology, Roswell Park Comprehensive Cancer Center, Buffalo, New York

**Keywords:** basal cell carcinoma, electrocoagulation, electrosurgery, Gorlin syndrome, Gorlin-Goltz syndrome, LC-OCT, line-field confocal optical coherence tomography, nevoid basal cell carcinoma syndrome

## Introduction

Gorlin syndrome, or nevoid basal cell carcinoma (BCC) syndrome, predisposes patients to the development of numerous BCCs over their lifetime, often beginning at a young age.^1^ In this population, routine biopsy of every clinically suspicious lesion may become impractical, especially for extremely small tumors.^2^ For such tiny lesions, biopsy is inherently burdensome because a disproportionately large amount of tissue may need to be removed relative to the size of the tumor in order to obtain an adequate specimen.^3^ Moreover, histopathologic confirmation requires processing time, delaying definitive treatment and often requiring the patient to return for another visit.^4^ When multiple tiny lesions are present, this can lead to repeated procedures, additional discomfort, and a substantial logistical burden. Over time, this repeated cycle of lesion detection, biopsy, treatment, and follow-up may contribute to substantial treatment fatigue in patients with nevoid BCC syndrome.

Noninvasive imaging may help address this challenge by enabling real-time lesion assessment at the point of care.^5^^,^^6^ Line-field confocal optical coherence tomography (LC-OCT) is particularly well-suited for evaluating small, clinically subtle keratinocyte carcinomas.^7^ We describe a patient with nevoid BCC syndrome who presented with multiple subtle suspicious papules, all of which were noninvasively confirmed as BCCs by LC-OCT and treated on the same day with electrocoagulation.

## Case report

**Fig 1:**
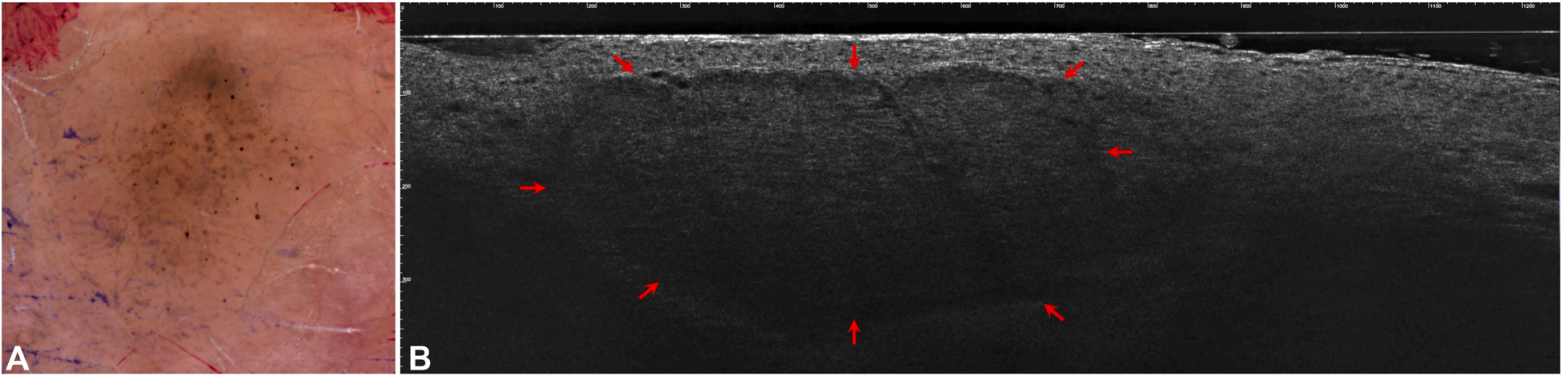
Pretreatment dermoscopic and line-field confocal optical coherence tomography features of basal cell carcinoma. **A,** Dermoscopic image of a representative lesion showing a structureless light brown to gray area with scattered fine *brown-black dots*, gray peppering, subtle shiny white structures, and absence of pigment network. **B,** Line-field confocal optical coherence tomography vertical section shows a hemispheric basal cell carcinoma lobule connected to the epidermis. The lobules show an inner grey core with a peripheral dark rim (*red arrows*).

A 66-year-old female patient with nevoid BCC syndrome presented to our dermatology clinic for evaluation of multiple new, clinically subtle papules on the head and neck region. The patient had a history of multiple BCCs, including clinically diagnosed BCCs previously treated with electrocoagulation with clinical clearance and no noted recurrence at those treated sites. On physical examination, 6 discrete lesions measuring 0.96 to 2.1 mm (mean: 1.5 mm) in diameter on dermoscopy were identified. The lesions presented as small, pink-to-skin-colored, faintly pigmented, flat to minimally elevated papules with ill-defined borders and no ulceration or crusting. On dermoscopic examination, the lesions demonstrated predominantly structureless light brown to gray areas with scattered fine brown-black dots and gray peppering, suggestive of BCC (Fig 1, *A*). Small focal blue-gray dots were present in some lesions, while classic BCC features such as well-formed blue-gray ovoid nests and arborizing vessels were notably absent. A pigment network was not observed, and cerebriform pattern, milia-like cysts, comedo-like opening, and hairpin vessels were also absent, ruling out the likelihood of a nevus or seborrheic keratosis. Subtle shiny white structures and fine surface scale were occasionally seen. These dermoscopy findings in the setting of basal cell nevus syndrome raised the suspicion of early BCCs.

Because of the high lesion count and the patient’s wish to minimize the burden of multiple biopsies, each lesion was evaluated with LC-OCT at the point-of-care. LC-OCT findings were consistent with BCC and included lobules with an inner gray core, a peripheral dark rim, and laminated architecture forming the characteristic millefeuille pattern (Fig 1, *B*). Across the 6 lesions, the imaging appearance was sufficiently concordant with BCC to confirm diagnosis and support immediate treatment. Lesions were well-demarcated on both dermoscopy and LC-OCT, and no breach of clinically defined margins was seen on LC-OCT.

After discussion of management options, all 6 lesions were treated on the same day with pinpoint electrocoagulation. For electrocoagulation, we used a Covidien Force FX electrosurgical generator in the “desiccate” setting at 10 Watts and performed precise monopolar pinpoint electrocoagulation of the targeted tissues. The patient tolerated the procedure well, with no immediate complications.

**Fig 2:**
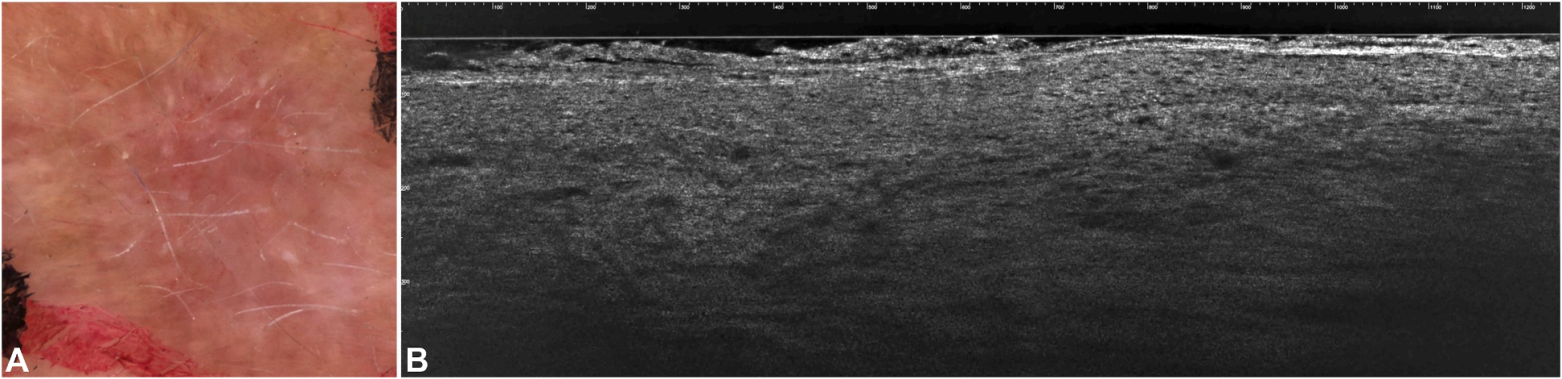
Dermoscopic and line-field confocal optical coherence tomography appearance 4 weeks after electrocoagulation. **A,** Dermoscopy shows a healed treatment site with mild background erythema, subtle whitish-pink fibrotic change and no residual BCC-specific structures. **B,** Corresponding line-field confocal optical coherence tomography image demonstrating only post-procedural inflammatory and fibrotic changes, without features of residual basal cell carcinoma. *BCC*, Basal cell carcinoma.

Four weeks after treatment, the patient returned for follow-up. The treated sites showed appropriate healing with no clinical evidence of persistence. Dermoscopy did not reveal any residual signs of BCC (Fig 2, *A*), and this was confirmed by LC-OCT, which demonstrated only post-treatment inflammatory and fibrotic changes secondary to electrocoagulation, without any features suggestive of residual BCC (Fig 2, *B*). Accordingly, no additional treatment was undertaken, and the treated sites will be monitored closely at routine follow-up visits.

## Discussion

Patients with nevoid BCC syndrome often develop numerous BCCs over time, making repeated invasive diagnostic procedures impractical and burdensome.^1^ In routine practice, the threshold for biopsy is often low, but when lesions are extremely small and numerous, a biopsy-based approach can become uncomfortable for the patient, time-intensive for the clinician, and cosmetically unfavorable, especially for very small tumors.^8^ This case illustrates how LC-OCT may be incorporated into the management of selected patients with multiple tiny (<2.5 mm) suspected BCCs. In our patient, LC-OCT supported the clinical and dermoscopic diagnosis, avoided 6 separate biopsies, and enabled same-day treatment of multiple clinically subtle BCCs. LC-OCT helped determine whether each lesion was sufficiently characteristic of BCC to proceed with treatment without immediate biopsy. This approach may reduce the need for histopathologic examination in carefully selected, low-risk lesions when clinical, dermoscopic, and LC-OCT findings are concordant. However, histopathologic confirmation may still become necessary when imaging features are equivocal. The short 4-week follow-up, and thus the absence of verification of durable clearance is an important limitation.

Building on prior use of LC-OCT for monitoring BCC response to systemic therapy,^9^ the practical advantage in this case was pairing noninvasive diagnosis and follow-up with a simple destructive treatment strategy. Electrocoagulation uses heat generated by an electric current to coagulate and ablate superficial tissue and may be suitable for selected tiny, well-demarcated, low-risk BCCs, such as superficial and early nodular subtypes, when recurrence or incomplete treatment risk is considered clinically tolerable.^10^ For carefully selected lesions in experienced hands, this approach may reduce the number of visits, biopsies, and interruptions to daily life for patients who require lifelong surveillance and hundreds of skin cancer treatments.

This case demonstrates a practical use of LC-OCT in a patient with nevoid BCC syndrome who developed 6 very small BCCs. Noninvasive confirmation followed by same-day electrocoagulation enabled efficient treatment while minimizing biopsy and visit burden.

## Conflicts of interest

None disclosed.
